# Statistical Report on Cataract

**Published:** 1871-09

**Authors:** E. L. Holmes

**Affiliations:** Professor of Ophthalmology and Otology in Rush Medical College; Surgeon to the Chicago Charitable Eye and Ear Infirmary


ARTICLE II.—*Statistical Report on Cataract.* By E. L. HOLMES, M.D., Professor of Ophthalmology and Otology in Rush Medical College; Surgeon to the Chicago Charitable Eye and Ear Infirmary. Read at the meeting of the Illinois Medical Society held at Peoria, May, 1871.

The operation for the removal of cataract has engaged the special study of many distinguished ophthalmic surgeons.

The wonderful and sudden change in the condition of the patient after a successful extraction, the difficulties attending its various steps, and the mental efforts required in its proper performance, have tended to attach unusual interest to this operation.

Leaving out of the estimate the statistics of a comparatively few operators in the whole world who have, as specialists, by long practice and an extraordinary repetition of the operation, acquired remarkable skill, we must look upon the extraction of cataract as a very difficult and dangerous operation; an operation which no one, however skillful in general surgery, should undertake without a special previous training. While saying this, I would disclaim all intention of throwing around the operation any mystery or unreal difficulties, which any true mechanic, I might say, with a delicate hand cannot overcome if he will devote himself long and earnestly to the subject.

I am certain the statistics of those who have been several years in practice, and yet operate scarcely more than six times in a twelve-month, would show a very large proportion of unfortunate results. I am impelled to make these remarks because the opinion is prevalent among our populace, and, I regret to say, among a part of our profession, that the removal of cataract is one of the simplest operations.

There is no difference of opinion among recognized authorities regarding the great superiority of the operation by extraction to that of couching. In fact, this last has become obsolete, and should be regarded as malpractice. The needle is almost absolutely reserved for infantile or very soft cataracts, especially in young persons. In older persons, even soft cataracts are usually extracted through a linear incision.

The two most important modifications in the operation for extraction are undoubtedly the removal of a portion of the iris, and the substitution, in place of the former flap incision, of a linear incision, a line or two posterior to the apparent union of the cornea and sclerotic—consequently in the sclerotic and conjunctiva; and yet, strictly speaking, the extremities of the incision alone lie in the sclerotic, the central portion lying in the cornea, although in that portion of it covered at its periphery by conjunctiva.

By enlarging the opening through the iris (iridectomy) any necessary introduction of instruments is less liable to injure the iris. The lens is extracted through the iris with much less danger of bruising or stretching it than through the normal pupil. Dilatation of the pupil, by means of atropine, instead of iridectomy, is of little benefit, since contraction almost invariably takes place as soon as the aqueous humor escapes.

The only especial difference in opinion regarding the iridectomy, is upon the question, whether the operation should be performed at the time of the extraction, or some weeks previous to it.

There are somewhat greater difficulties in performing the iridectomy with a large wound in the conjunctiva than in the ordinary manner. The large wound in the sclerotic and iris made at the same time, would seem to increase the danger of inflammation.

The fact that the very few operators who extract a large number of cataracts annually, have a very high proportion of good results by the more speedy method, is not an argument for its adoption by those who operate less frequently.

The advantage derived from the modification in the form, and especially the position of the incision, is certainly great. This form of incision gives the largest opening, with the least possible wound of tissue.

The wound through the sclerotic and vascular conjunctiva heals more readily than when made through the cornea proper. The wound in the sclerotic is in the same relative condition as a subcutaneous incision in other operations, for the conjunctiva usually heals with great rapidity.

I cannot, in any way, give you a better idea of this incision than you can obtain by inspecting this hollow model of the eye, with the transparent cornea and pupil in their relative size and position.

The curved white line on the true cornea, less than a line within the corneal border, represents the incision in the former flap operation. The other line is external to the cornea proper, and is drawn in such a way that the centre lies very near the periphery of the cornea, while the extremities are each about a quarter of an inch (on the enlarged model) from it.

To those who have seen only the old flap operation, so perfectly bloodless, the operation as above described will appear a barbarous procedure.

I do not wish, at this time, to deliver a lecture upon the modified peripheral extraction. For a full description of the manner in which the lids and globe are controlled, in which the capsule is ruptured, the lens extracted, and the compressive bandage applied, I must refer you to the papers of Græfe, and the various articles which have appeared in our later text books and journals. The monograms of Græfe are classical, and should be most carefully studied by all who would attempt to operate for cataract.

During the past fourteen years I have examined 8,967 patients affected with diseases of the eye. Of this number, 243 were suffering from some abnormal condition of the lens.

The following is a table of the diseases and injuries of the lens, and the operations performed:

DISEASES.

| | |
|---|---|
| Cataract Incipient, | 29 |
| " Hard, | 72 |
| " Soft, | 26 |
| " Pyramidal, | 1 |
| " Congenital, | 28 |
| " Traumatic, | 32 |
| " Central Layer, | 13 |
| Dislocation of Lens into Anterior Chamber, | 7 |
| | 208 |

| | |
|---|---|
| | 208 |
| Dislocation of Lens into Vitreous, - - - - - - | 4 |
| Dislocation Spontaneous into Anterior Chamber, - - - - | 2 |
| Dislocation Spontaneous into Vitreous, - - - - - - - | 4 |
| Capsular Remains, - - - - - - - - - | 19 |
| Deposits on Capsule, - - - - - - - - - | 4 |
| Foreign Body (steel) in Lens—no Inflammatory Action, - - | 2 |
| | 243 |

OPERATIONS.

| | |
|---|---|
| Extraction Flap, - - - - - - - - - | 7 |
| " " (preliminary iridectomy), - - - - - | 12 |
| Extraction Linear, - - - - - - - - - - | 9 |
| " " (preliminary iridectomy), - - - - - | 3 |
| Extraction Græfe's Modified Linear, - - - - - - | 46 |
| Depression of Lens, - - - - - - - - - | 2 |
| Division (cornea), - - - - - - - - - - | 14 |
| " (sclerotic) - - - - - - - - - | 11 |
| Removal of Capsular Remains, - - - - - - - | 16 |
| | 120 |

Regarding the result of operations performed when I had little experience, I can only refer to the report I presented at the last meeting.

Of the forty-six extractions through the modified linear section, six were total failures, and forty with good results. I may remark, that of the forty successful cases, thirty-two in succession were successful, with scarcely an accident during the operation, or pain after it. Of these favorable cases, two were complicated with adhesions of the iris, two with solution of the vitreous humor, and four with sequelæ of chronic conjunctivitis.

Of the six unfortunate cases, two were complicated with extensive posterior synechia and two with serious sequelæ of conjunctivitis. All these unfavorable results were due to iritis, except one. In a single patient there was a fearful suppurative irido-choroïditis.

Of the accidents which may justly be charged to the operator, and yet are perhaps in some cases unavoidable, I may mention two cases, in which, from the great hardness of the cortical substance, the incision was found too small, and was necessarily enlarged.

In one case, in which I had supposed the cataract was hard, the lens was found to be composed of quite a thick capsule, filled with an opaque fluid, which readily escaped on rupturing the capsule.

In four cases the lens was delivered with the capsule intact.

In one case the conjunctiva was so firmly stretched over the sclerotic that it was impossible to grasp it with the forceps; in two others it was so excessively loose and flaccid, that there was difficulty in preventing a large fold of conjunctiva from being crowded over the cornea. In three operations considerable vitreous was lost, and in five a minute portion.

In thirteen cases there were extensive capsular remains, in four of which I removed a portion of the capsule, subsequently with great improvement of sight; in two, vision would undoubtedly have been greatly increased if the patients had been in circumstances to admit of the operation; in the other seven cases, the membrane was so thin, covering but a part of the pupil, that operative interference seemed scarcely demanded.

I had been so fortunate in the removal of several capsular remains left after the use of the needle by unskillful operators, that I had felt less fear of evil consequences than the nature of the operation really demands. I have removed several by means of the iris-hook, three with the hook aided by a stop-needle, and three through an incision in the sclerotic, as made in Hancock's operation. In only one case did any serious inflammation arise in removing the capsule. In this single case, a young man with congenital opaque lens, had a thick capsular membrane, subsequent to an operation years before with a needle in the hands of a traveling charlatan. I removed it through an opening (Hancock's) in the sclerotic. Severe inflammation arose, finally causing atrophy of the globe.

I may here state that I have never, in but one instance, operated on both eyes at the same sitting.

After numerous trials, I have found that I could hold the globe more steadily with the forceps by seizing the conjunctiva nearer the point where the knife enters the eye, than further from this point, as is usually advised. I prefer this position, especially where the conjunctiva is very loose and movable.

I believe, in cases in which after rupture of the capsule there is difficulty in causing the lens to advance towards the opening in the globe, there is less real violence in "delivering" the cataract at once, by the cautious use of a suitable hook, than in protracted, though gentle, manipulation. Previous to the appearance of Græfe's paper, I had already, in several cases (preliminary iridectomy) where the cataract did not pass readily into the wound, used a blunt hook, introduced behind the lens, and, as it appeared, with exceedingly gentle force, removed the lens with the most satisfactory results. As I almost invariably give the patient ether, there is less danger of injury by the use of the hook than where an anæsthetic is not given. It should be borne in mind, however, that instruments should be inserted as rarely and as gently as possible within the globe.

In regard to the compressive bandage, which is applied to secure coaptation of the edges of the wound, I must say I have secured a more even and more easily adjusted pressure, by the use of a thin band of linen, somewhat worn, reaching nearly around the head, the interval (posteriorly) being filled with elastic rubber. The pressure produced by this band can be decreased by cutting into the rubber, or increased by taking a few stitches in the linen. In dressing the eye, it can be readily slipped upon the forehead, and as readily readjusted over the compresses.

I have generally extracted by the lower section, because there is ordinarily less difficulty, and because, so far as I can judge by comparison of my cases, and those of other operators in whom the upper section had been made, I have not observed that vision was better than in those in whom the extraction was by the lower section.

Regarding the acuteness of vision in the cases here reported, I cannot present a correct tabular statement, since nearly all the successful cases left my care before the eighteenth day, two before the tenth day, after the operation. They nearly all lived at a distance, and subsequently sent to the optician for a series of lenses from which to make a selection. Three cases, I know, were quite astigmatic; vision was improved by tilting the lens.

I know that several of these patients are able to write and read easily ordinary print; two to sketch accurately fine drawings, and nearly all to support themselves.

